# Zinc Substitution of Cobalt in Vitamin B_12_: Zincobyric acid and Zincobalamin as Luminescent Structural B_12_‐Mimics

**DOI:** 10.1002/anie.201908428

**Published:** 2019-09-04

**Authors:** Christoph Kieninger, Joseph A. Baker, Maren Podewitz, Klaus Wurst, Steffen Jockusch, Andrew D. Lawrence, Evelyne Deery, Karl Gruber, Klaus R. Liedl, Martin J. Warren, Bernhard Kräutler

**Affiliations:** ^1^ Institute of Organic Chemistry and Center for Molecular Biosciences (CMBI) University of Innsbruck 6020 Innsbruck Austria; ^2^ School of Biosciences University of Kent Canterbury CT2 7NJ UK; ^3^ Institute of General, Inorganic and Theoretical Chemistry and Center for Molecular Biosciences (CMBI) University of Innsbruck 6020 Innsbruck Austria; ^4^ Department of Chemistry Columbia University New York USA; ^5^ Institute for Molecular Biosciences University of Graz Austria

**Keywords:** cobalamins, photochemistry, transition metal, vitamin, X-ray structure

## Abstract

Replacing the central cobalt ion of vitamin B_12_ by other metals has been a long‐held aspiration within the B_12_‐field. Herein, we describe the synthesis from hydrogenobyric acid of zincobyric acid (**Znby**) and zincobalamin (**Znbl**), the Zn‐analogues of the natural cobalt‐corrins cobyric acid and vitamin B_12_, respectively. The solution structures of **Znby** and **Znbl** were studied by NMR‐spectroscopy. Single crystals of **Znby** were produced, providing the first X‐ray crystallographic structure of a zinc corrin. The structures of **Znby** and of computationally generated **Znbl** were found to resemble the corresponding Co^II^‐corrins, making such Zn‐corrins potentially useful for investigations of B_12_‐dependent processes. The singlet excited state of **Znby** had a short life‐time, limited by rapid intersystem crossing to the triplet state. **Znby** allowed the unprecedented observation of a corrin triplet (*E* ^T^=190 kJ mol^−1^) and was found to be an excellent photo‐sensitizer for ^1^O_2_ (Φ_Δ_=0.70).

## Graphical abstract

**Zincobyric acid and zincobalamin**, the Zn‐analogues of cobyric acid and of vitamin B_12_, were efficiently prepared from the metal‐free corrin, hydrogenobyric acid. The crystal structure of the zincobyrate, the first of a Zn‐corrin, revealed a significant misfit of the closed shell Zn^II^‐ion to the corrin ligand. The luminescent Zn‐mimics of vitamin B_12_ do not support organometallic B_12_‐enzyme reactions and may be useful enzyme inhibitors.

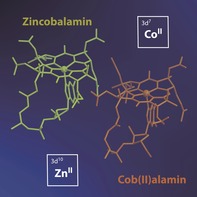

The biological use of cobalt as the specific transition metal center in natural B_12_‐cofactors and the interaction between cobalt and the corrin ligand raise intriguing questions concerning the origins of its natural selection.^1^ Engineered B_12_‐biosynthesis^2^ has opened up a preparative route to hydrogenobyric acid (**Hby**),^3^ the metal‐free corrin ligand of vitamin B_12_, providing an excellent opportunity for the synthesis of transition‐metal analogues of the natural cobalt‐corrinoids.^4^ Zn^II^‐analogues of natural corrinoids have hardly been explored4b but are attractive, as Zn‐ and low‐spin Co^II^‐centers exhibit similar structural properties in small complexes and in metalloproteins.^5^

Fischli and Eschenmoser reported the synthesis and characterization of the first Zn‐corrin (**ZnCor**), when exploring the synthesis and chemistry of corrins in model studies towards the total synthesis of vitamin B_12_.4a, 6 Indeed, in the Eschenmoser^7^ and Woodward labs^8^ a 5,15‐nor‐zincobyrinate was an intermediate of the B_12_‐synthesis. UV/Vis‐spectroscopically characterized samples of zincobalamin (**Znbl**) and zincobyric acid (**Znby**), the Zn‐analogues of vitamin B_12_ (**CNCbl**) and cobyric acid (**Cby**) (Scheme 1), were first reported by Koppenhagen and Pfiffner.^9^

**Scheme 1:**
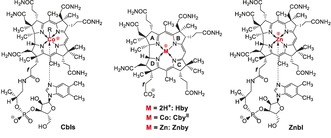
Formulae of metal‐free, cobalt‐ and zinc‐corrinoids. Left: General formula of the cobalamins vitamin B_12_ (R=CN, **CNCbl**), coenzyme B_12_ (*R*=5′‐deoxyadenosyl, **AdoCbl**), cob(II)alamin (R=e^−^, **Cbl^II^**) Center: Formulae of hydrogenobyric acid (**Hby**), Co^II^‐cobyric acid (**Cby^II^**) and zincobyrate (**Znby**), where the axial solvent ligands for both the Zn^II^ and Co^II^ have been omitted. Right: formula of zincobalamin (**Znbl**) in its “base‐on” form.

Herein, we delineate an effective synthesis of **Znby** and of **Znbl**, starting from crystalline **Hby**,^3^ describe the pertinent spectroscopic and structural properties of these luminescent B_12_‐derivatives and report a kinetic study of the binding of Zn^II^‐ions to **Hby. Znby** was prepared at room temperature in 83 % yield from **Hby** 3 and Zn^II^acetate (see Scheme 2 and the Supporting Information). Zn^II^‐ions bound to **Hby** readily under these conditions (Supporting Information, Figure S4), and over 20 times faster than Co^II^‐ions. **Znby** was resistant to removal of the Zn^II^‐ion in acidic aqueous solution, and **Hby** could not be efficiently (re)generated from **Znby**.

**Scheme 2:**
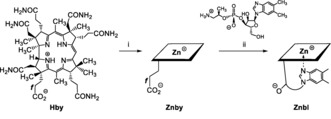
Preparation of **Znby** and **Znbl** from **Hby**. i) 2 mg **Hby** in 2.3 mL aq. 0.5 mm Zn(OAc)_2_ at pH 6, 80 min, room temperature; ii) 5 mg **Znby**, 3.3 meq B_12_‐nucleotide moiety, 20 meq HOBt and 23 meq EDC*HCl in 1.9 mL H_2_O, 4 h, 0 °C (see the Supporting Information for details).

The UV/Vis spectrum of **Znby** in aqueous buffer, pH 5, displayed absorption maxima at 335 nm, 493 nm, and 518 nm9a (see Figure 1) and showed similar basic features as those recorded for **ZnCor** 6a and for a 5,15‐nor‐zincobyrinate,^7^, ^8^ but with maxima at roughly 20 nm longer wavelengths. The aqueous solution of **Znby** fluoresced with a maximum emission at 552 nm.

**Figure 1:**
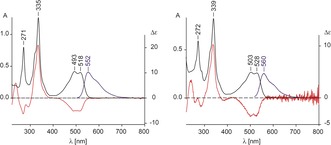
Absorption and fluorescence spectra of **Znby** and **Znbl** at 298 K. Left: UV/Vis absorption (black trace), CD (red trace), and fluorescence emission (blue trace) of **Znby** in H_2_O. Right: UV/Vis absorption (black trace), CD (red trace), and fluorescence emission (blue trace) of **Znbl** in 10 mm Na‐phosphate buffer, pH 5 (see the Supporting Information for details).

The solution structure of **Znby** (molecular formula C_45_H_64_N_10_O_8_Zn, see Supporting Information, Figure S3) was characterized by NMR spectroscopy, providing assignment of 52 H‐atoms and of all C‐atoms (Supporting Information, Table S1). A 500 MHz ^1^H‐NMR spectrum of **Znby** in D_2_O (Figure 2 a) featured eight methyl singlets, the singlet of HC10 at 5.51 ppm, and signals for HC19, HC3, HC8, and HC13 at intermediate field.

**Figure 2:**
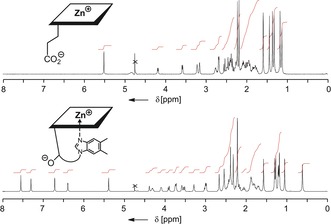
500 MHz ^1^H‐NMR spectra of **Znby** and **Znbl** (in D_2_O, 298 K). Top: **Znby** (*c*=1.1 mm). Bottom: **Znbl** (*c*=7.2 mm); residual water signal after pre‐saturation marked by an X.

Covalent attachment of the B_12_‐nucleotide moiety1a, 10 to **Znby** was achieved using a recently developed carbodiimide method.2d, 11 In brief, from 5.0 mg (4.8 μmol) of **Znby** 4.8 mg of **Znbl** (3.6 μmol, 75 %) were obtained, after chromatography and crystallization from aqueous acetonitrile (Scheme 2). An aqueous solution of **Znbl** exhibited a UV/Vis spectrum as previously reported9b (Figure 1). The absorption maxima of the α,β‐bands in the UV/Vis spectrum of **Znbl** occurred at 528 and 502 nm, suggesting intramolecular coordination of the nucleotide base.4b A fluorescence spectrum of **Znbl** showed an emission with a maximum at 560 nm, that is, about 8 nm longer wavelength than in the spectrum of **Znby** (Figure 1). The structure of **Znbl** (molecular formula C_62_H_88_N_13_O_14_PZn, see Supporting Information, Figure S5) was established by NMR spectroscopy (Figure 2 and Supporting Information, Table S2), providing assignment of 73 H‐atoms and all C‐atoms. The high‐field shifts of the signals of H_3_C1A (by about 0.5 ppm to *δ*=0.65 ppm) and of HN2 and HCN7 of the DMB‐moiety, by about 0.8 ppm to *δ*=7.55 ppm and *δ*=6.72 ppm, respectively, indicated a “base‐on” form, as in Co^III^cobalamins^12^ and in Co^II^cobalamin (**Cbl^II^**). The intramolecular Zn‐coordination of the DMB‐base was analyzed further using ^1^H,^1^H‐ROESY spectroscopy (see Supporting Information, Figure S3), characterizing **Znbl** as a roughly *iso*‐structural analogue of **Cbl^II^**.^13^

**Znby** furnished orange‐red single crystals from aqueous acetonitrile (*P*2_1_2_1_2_1_), suitable for X‐ray crystal structure analysis (Figure 3 and Supporting Information, Table S3). The Zn^II^‐center is coordinated in an approximate pyramidal fashion, where the axial ligand is attached to the “top” (or β) face of the corrin‐bound Zn‐ion, lifting it by 0.624 Å from the best plane through the inner corrin N‐atoms (Figure 3). However, in the crystal the individual **Znby**‐molecules were part of a coordinative **Znby** polymer, generated by repetitive intermolecular axial Zn^II^‐coordination by the carboxylate function of a neighboring **Znby** molecule (see the Supporting Information).

**Figure 3:**
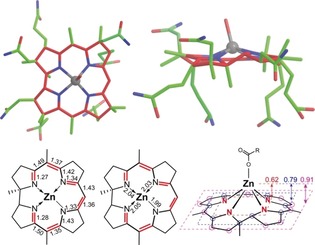
Top: X‐ray crystal structure of **Znby** in two projections (color coding: red=corrin core carbons and oxygens; green=other carbons; blue=nitrogens; gray=Zn). Bottom: Simplified formulae with lengths of corrin π‐bonds and Zn−N bond lengths, coordination geometry around Zn‐center and “doming” of the corrin ligand (represented by the distances of the Zn‐ion from the best planes through the coordinating N‐atoms (red label), the adjacent C‐atoms (blue label), and the further C‐atoms (pink label)).

A comparison of the crystal structures of **Znby** and **Hby** 3 (Figure 4 and Supporting Information, Table S4) indicates a minor increase only in the radial size of the coordination hole on Zn‐binding. The average lengths of the N1–N3 and N2–N4 diagonals in **Hby** (*d*=3.82 Å) and in **Znby** (*d*=3.84 Å) are similar. Coordination of Zn^II^ leads to a reduction of the corrin “helicity” *h* from *h*=12.9° in **Hby** 3 to *h*=8.0° in **Znby** (Supporting Information, Table S4). The major effects of the formal replacement of the penta‐coordinate Co^II^‐center in a vitamin B_12_ derivative by a Zn^II^‐ion are seen in a structural comparison of **Znby** and Co^II^‐heptamethyl‐cobyrinate perchlorate (**Cbin^II^**)^14^ (Figure 4 and Supporting Information, Table S4). The Zn−N bonds in **Znby** (average length=2.03 Å) are longer than those found in **Cbin^II^** (average Co−N bond length=1.90 Å). Likewise, the axial displacement of the metal‐ion from the mean plane through the four corrin N‐atoms in the Zn‐corrinate **Znby** (0.624 Å) is palpably greater than that of the Co^II^‐center of **Cbin^II^** (0.048 Å). In **Znby** and **Cbin^II^**, an axial ligand is bound at the β‐face with a long metal‐oxygen bond, and the four corrin N‐atoms are displaced slightly from a planar to a squashed tetrahedral arrangement (Supporting Information, Table S4). However, whereas the core of the corrin ligand is made nearly C2‐symmetrical by the coordination of a Co^II^‐center, in **Znby** the N2–N4 diagonal remains remarkably longer than its N1–N3 counterpart, with Δ*d*=0.186 Å. Hence, about 60 % of Δ*d*=0.297 Å in **Hby** are retained in the corrin ligand of **Znby**. This feature of **Znby** reflects a preferred mode of the conformational adaptation of the coordination hole of the flexible, unsymmetrical corrin ligand to the 5‐coordinate closed‐shell Zn‐ion. The “helicity” *h*(**Znby**)=8.0° is in line with a small directional effect of Zn^II^, compared to Co^II^‐ or Co^III^‐binding, where *h*=6.1° in **Cbin^II^** and *h*=4.1° in **CNCbl**.^3^ In **Znby**, the corrin ligand adapts to the skewed pyramidal arrangement around the Zn^II^‐center by an unprecedented conformational “doming” of the corrin ligand (Figure 4). Consequently, the corrin‐based inter‐planar angle *ϕ* 3 of the coordination polyhedral at the Zn‐center *ϕ*(**Znby**)=50.2° far exceeds *ϕ*(**Cbin^II^**)=7.6° and *ϕ*(**CNCbl**)=4.6°.^3^

**Figure 4:**
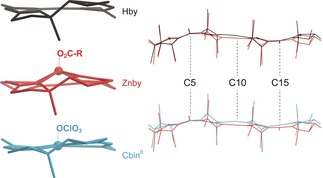
Left: Comparison of crystal structures of **Znby**, **Hby**, and **Cbin^II^**. Right: Superposition of the cylindrical projections (top) of **Znby** (red) and **Hby** (black) and (bottom) of **Znby** (red) and **Cbin^II^** (blue).

The fluorescence of **Znby** in EtOH at 296 K showed an emission maximum at 548 nm and an energy of the lowest singlet excited state of **Znby** of 225 kJ mol^−1^, close to the value observed with the metal‐free **Hby** (*E* ^S^=223 kJ mol^−1^).^3^ Hence, the closed shell Zn‐ions do not appear to significantly perturb the π,π*‐transitions of the corrin ligand. However, the fluorescence of **Znby** (fluorescence lifetime *τ*_f_<0.4 nsec) decayed about an order of magnitude more rapidly at 23 °C than that of **Hby** (*τ*_f_=3.3 nsec), exhibiting a correspondingly lower quantum yield Φ_f_=0.025 (for **Hby** Φ_f_=0.18). The short fluorescence lifetime of photo‐excited **Znby** at 296 K is due to the efficient singlet‐triplet intersystem crossing with an estimated rate of more than 2×10^9^ sec^−1^, boosted by the coordination of the Zn‐ion.^15^ At 77 K the solution of **Znby** in EtOH displayed an absorption maximum at 523 nm, and emitted both fluorescence (*λ*_max_=538 nm) and phosphorescence (first maximum at 628 nm, Figure 5, see the Supporting Information for details). Hence, at 77 K the lowest triplet state of **Znby** occurred at *E* ^T^=190 kJ mol^−1^, furnishing the first such benchmark value for a natural corrin ligand. The phosphorescence of photo‐excited **Znby** decayed with a lifetime of 13±1 msec at 77 K. **Znby** sensitized the formation of ^1^O_2_ with a quantum yield Φ_Δ_=0.70. The Zn‐corrin **ZnCor** 6a emitted fluorescence with a maximum at 573 nm (Φ_f_=0.09) at room temperature in EtOH,^16^ and was an efficient triplet sensitizer in the legendary photo‐induced A/D‐secocorrin to corrin cycloisomerization.4a, 7, 16

**Figure 5:**
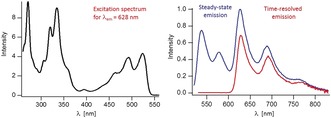
Phosphorescence excitation (left) and emission spectra (right) of **Znby** in EtOH at 77 K. The excitation spectrum (left) was recorded by monitoring phosphorescence at 628 nm. The steady‐state emission spectrum (right, blue line) was recorded with excitation at 515 nm, featuring both, the fluorescence and phosphorescence of **Znby**. The time‐resolved phosphorescence spectrum of **Znby** (right, red line) was recorded 2–12 msec after the pulsed excitation at 528 nm.

To shed further light on the structure of **Znby**, the gas‐phase structure of the hypothetical 4‐coordinate analogue **Znby(4)** was calculated, using DFT, from the crystal structures of **Hby**, as well as of the heptamethyl ester **Cbin^II^**, the latter providing computational **Znby** models in which the polar side chain functions are replaced by methyl ester groups (for details, see the Supporting Information). Ligation of an acetate ligand at the “upper” (β) or at the “lower” (α) side of the latter **Znby(4)** structure, furnished models of **Znby** and of its coordination isomer **Znby(α)**. The calculated structure of **Znby** closely reflected the observed crystallographic structural peculiarities of **Znby**, such as the longer N2–N4 diagonal (Δ*d*_calc_=0.22 Å), the long Zn−N‐bonds (Zn−N_av_=2.06 Å), the out‐of‐plane position of the 5‐coordinate Zn‐ion (0.65 Å), and the doming of the corrin ligand. In **Znby(α)**, the calculations generated a model with comparably long Zn−N‐bonds (Zn−N_av_=2.06 Å), an N2–N4 diagonal shorter than N1–N3 (Δ*d*_calc_=−0.10 Å), a profound out‐of‐plane position of the 5‐coordinate Zn‐ion (−0.62 Å) and an “inverted” doming of the corrin ligand. A structure of **Znbl** was calculated (Figure 6) starting from a previously optimized gas‐phase structure of **Cbl^II^**. It showed a pronounced out‐of‐plane movement of the 5‐coordinate Zn‐ion (−0.46 Å), exceeding that of Co^II^ in **Cbl^II^** (−0.13 Å), but compensated in part by the slightly shorter Zn−N_DMB_‐bond (2.07 Å) in **Znbl** than the Co−N_DMB_ bond (2.11 Å) in **Cbl^II^**. The structure of **Znbl** showed a downward movement of the DMB‐base, compared to **Cbl^II^**, but was similar in its overall architecture. Hence, **Znbl** can be considered as a good structural mimic of the non‐luminescent **Cbl^II^**.

**Figure 6:**
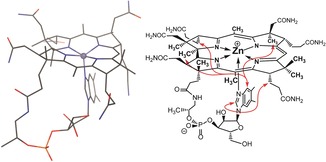
The “base‐on” structure of **Znbl**, calculated by DFT (left), and from NMR‐derived correlations between corrin and DMB‐ moieties (right).

As an *iso*‐structural analogue of some **Cbl**s that is inactive in the organometallic processes typical of B_12_‐dependent enzymes, **Znbl** may represent an “antivitamin B_12_”^17^ and be a useful fluorescent molecular probe in B_12_‐biology and biomedicine.^18^ The structure analysis of **Znby** has indicated that the closed shell d^10^‐ion of Zn^II^ lacks the precise fit of the similarly sized low‐spin Co^II^‐centers (d^7^‐ions),^19^ where an empty d_x_^2^−_y_^2^‐orbital provides an excellent electronic complement for the four corrin N‐atoms.^20^ Hence, the basic fit of low spin Co^II^‐ and diamagnetic Co^III^‐ions to the ring size of the corrin ligand^3^ is not extended to the 5‐coordinate Zn^II^‐ion. A similar (but less pronounced) difference is seen in Zn^II^‐ and Co^II^‐porphyrins, where porphyrin “doming” and axial displacement of 5‐coordinate Zn^II^‐centers towards the axial ligand exceed the effect of the 5‐coordinate Co^II^‐ions.^21^

The lack of out‐of‐plane displacement of the 5‐coordinate Co^II^‐centers in Co^II^‐corrins appears to be a consequence of the partially occupied valence shell of this electronically adaptable d^7^‐ion. Indeed, the 15‐membered equatorial perimeter of the “ring contracted” corrin ring is able to accommodate the size of both low‐spin Co^II^‐ and diamagnetic Co^III^‐ions, which have the capacity to fit their electronic configuration to favorable interactions with the ligand.4a, 5a, 5b, 22 In contrast, when binding a 5‐coordinate closed shell d^10^ Zn^2+^‐ion, the corrin ligand undergoes doming and further conformational relaxations. In spite of the structural differences between **Znby** and **Cbin^II^**, as well as those deduced for **Znbl** and **Cbl^II^**, the redox‐inactive Zn‐complexes of natural corrins may be useful as luminescent (inactive) mimics of corresponding B_12_‐derivatives.

The work reported here describes a rational avenue to the construction and characterization of **Znbl**, promising to be useful in biological and biomedical experiments. Significantly, the engineering of bacterial strains for the production **Hby** 3 has unlocked the gateway to the direct generation of a range of other **Metbl**s and **Metby**s, the transition‐metal analogues of the **Cbl**s and **Cby**s, respectively. The helical, un‐symmetric natural corrin‐ligand is a unique binding partner for transition‐metal ions, providing an exciting opportunity to construct a diverse range of metal analogues of vitamin B_12_, investigate their structural behavior, examine their reactivity, and to test biological effects.

## Experimental Section

*Crystallographic Data*. X‐ray crystal data of **Znby** have been deposited at the Cambridge Crystallographic Data Centre (CCDC) under the reference number CCDC https://www.ccdc.cam.ac.uk/services/structures?id=doi:10.1002/anie.201908428.

## Conflict of interest

The authors declare no conflict of interest.

## Supporting information

As a service to our authors and readers, this journal provides supporting information supplied by the authors. Such materials are peer reviewed and may be re‐organized for online delivery, but are not copy‐edited or typeset. Technical support issues arising from supporting information (other than missing files) should be addressed to the authors.

Supplementary
