# Supplementary material for: Zinc Substitution of Cobalt in Vitamin B12: Zincobyric acid and Zincobalamin as Luminescent Structural B12‐Mimics
Source: Angew Chem Int Ed Engl. 2019 Sep 4;58(41):14568–72. doi: 10.1002/anie.201908428 (PMC6790578; doi:10.1002/anie.201908428)
Supplement: Supplementary file 1 — Supplementary [file ANIE-58-14568-s001.pdf]

Angewandte Chemie
Eine Zeitschrift der Gesellschaft Deutscher Chemiker

Supporting Information

Zinc Substitution of Cobalt in Vitamin B_{12}: Zincobyric acid and Zincobalamin as Luminescent Structural B_{12}-Mimics

Christoph Kieninger[+], Joseph A. Baker[+], Maren Podewitz[+], Klaus Wurst, Steffen Jockusch, Andrew D. Lawrence, Evelyne Deery, Karl Gruber, Klaus R. Liedl, Martin J. Warren, and Bernhard Kräutler**

anie_201908428_sm_miscellaneous_information.pdf

Supporting Information

Table of Contents

1. Materials 2
2. Spectroscopy and Instruments 2
3. Synthesis, purification and spectral analysis of zincobyric acid 2
4. Synthesis, purification and spectral analysis of zincobalamin 6
5. Crystallization of zincobyric acid and single-crystal X-ray structure analysis 10
6. Metal incorporation into hydrogenobyric acid – kinetic analysis 13
7. Photo-physical measurements of zincobyric acid 14
8. Computational structure calculations of zincobyric acid 15
9. References 21

1. Materials

B_{12}-Nucleotide was synthesized according to[1]; Methanol (MeOH), acetonitrile (MeCN), HiPerSolv Chromanorm and acetic acid (HOAc) p.A., sodium hydroxide (NaOH) p.A. from VWR chemicals; zinc acetate ($Zn(OAc)_2$) p.A. from Acros Organics; tetrafluoroboric acid 48% in H_2O from Sigma Aldrich; sodium tetrafluoroborate, p.A., sodium acetate (NaOAc); sodium dihydrogenphosphate (NaH_2PO_4), disodium hydrogenphosphate (Na_2HPO_4), 1-hydroxybenzotriazole (HOBt) purum, potassium cyanide p.A. (KCN) from Fluka; N-(3-dimethylaminopropyl)-N′-ethylcarbodiimide hydrochloride (EDC*HCl) 98% from Alfa Aesar; water purified by reversed osmosis and MilliQ academic system; D_2O 99.96%D from Eurisotop; Sep-Pak® C18 cartridges (various sizes, conditioned with 20 mL MeOH and 60 mL H_2O prior to use) from Waters;

2. Spectroscopy and Instruments

UV-Vis: Agilent Cary 60. CD: Jasco J-715 spectro-polarimeter. Fluorescence: HORIBA Jobin Yvon Fluorolog-3 (steady-state fluorescence spectra); Edinburgh Analytical Instruments OB920 (fluorescence lifetimes). Singlet oxygen phosphorescence: HORIBA Jobin Yvon, modified Fluorolog-3 spectrometer in conjunction with a NIR sensitive photomultiplier tube (Hamamatsu, H10330A-45). Spectra Physics GCR-150-30 Nd:YAG laser (355nm, ca. 5mJ per pulse, 5ns) was used for pulsed excitation to collect the singlet oxygen phosphorescence decay traces at 1270 nm on a digital oscilloscope (Tektronics, TDS 360). NMR: 500 MHz Varian Unity Inova, 5mm triple-resonance probe with z-gradients, puls sequences from VNMRJ-ChemPak 4.1; ^1H reference to δ(HDO) = 4.75 ppm, chemical shift and signal assignment was based on ^1H, (^1H,^1H)-COSY, (^1H,^{13}C)-HSQC, (^1H,^{13}C)-HMBC and (^1H,^1H)-ROESY spectra; ESI-HR-MS: Thermo Scientific LTQ-Orbitrap XL, (+)-ion mode, 4.5 kV. HPLC: Hitachi Elite LaChrom, L2130 pump, L245 diode array detector; Dionex Ultimate 3000, variable wavelength detector; column: YMC-Triart –C18, 250x4.7 mm, S-5 µm, 12 nm; solvent composition: A: 10 mM aqueous NH_4OAc pH 7, B= MeOH; 8% to 95%B 0-40 min, 95% B 40-44 min, 95% to 8%B 44-45 min, flow= 1 mL/min.

3. Synthesis, purification and spectral analysis of zincobyric acid

All operations were performed with protection from light.

In a 5 mL glass vial 2.0 mg (2.3 µmol) crystalline hydrogenobyric acid (**Hby**)[2] were dissolved in 2.3 mL 0.5 M $Zn(OAc)_2$ pH 6 and stirred for 80 min at RT. The reaction solution was diluted with H_2O to 15 mL and loaded on a Sep-Pak® C18 Classic Cartridge. The adsorbed **Znby** was washed with 20 mL H_2O followed by 20 mL 100 mM $NaBF_4$ aq. pH 6 and 20 mL H_2O. The **Znby** was eluted with 3 mL 100 µM $NaBF_4$ in MeOH and the solvents were evaporated in Ar-stream at RT. The solid **Znby** was dissolved in 50µL H_2O and ~200 µL MeCN. The **Znby** was crystallized by

slow addition of ~1 mL MeCN, storage at RT for 3 h, and at 5±3°C overnight. Additional 2 mL MeCN were added and the suspension was stored for further 24h at 5±3°C. The mother liquor was separated, the crystals were washed with 2 x 1 mL MeCN and dried in HV for 6 h. 1.3 mg (1.4 µmol, 61%) crystalline **Znby** was obtained as orange crystals. Further 0.5 mg (0.5 µmol, 22%; total: 1.8 mg, 1.9 µmol, 83 %) of **Znby** were obtained from the mother liquor.

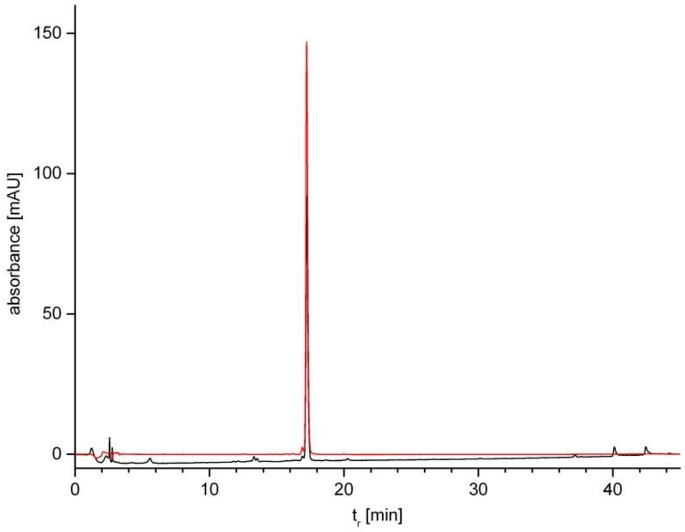

Figure S1. HPLC chromatogram of crystalline **Znby** (black trace λ=280nm, red trace λ=520nm)

UV-Vis (c = 18.8 µM in H_2O, RT): λ^{max} [nm] (log ε) = 235 (4.03), 271 (4.63), 323 (sh, 4.58), 335 (4.83), 357 (sh, 4.08), 396 (3.32), 468 (sh, 4.13), 493 (4.34), 518 (4.33) (see main text, Figure 1)

CD (c = 39.0 µM in H_2O, 293K): $\lambda^{max/min}$ [nm] (Δε [$l*mol^{-1}*cm^{-1}$]) = 236 (6.0), 269 (-6.3), 281 (-2.6), 291 (-3.5), 322 (sh, 12.2), 334 (21.1), 490 (-5.2), 518 (-5.2); λ^0 [nm] = 248, 305, 400 (see main text, Figure 1)

Fluorescence (c = 6.44 µM in H_2O, RT):
emission spectrum (excitation at 495 nm): λ^{max} [nm] (I) = 552 (193), 580 (sh, 138)
excitation spectrum (emission at 552 nm): λ^{max} [nm] (I) = 272 (248), 324 (sh, 265), 335 (349), 493 (209), 518 (226) (see main text, Figure 1)

High resolution ESI-MS (ESI-LTQ-Orbitrap, MeOH, m/z≥400): m/z (%) = 991.399 (6, $[M+2O+Na]^+$); 973.413 (6), 972.418 (6), 971.415 (11), 970.421 (12), 969.418 (23, $[M+2O+H]^+$); 942.428 (20), 941.425 (42), 940.428 (41), 939.426 (58), 938.431 (62), 937.428 (100, $[C_{45}H_{65}N_{10}O_8Zn]^+$ ≡ $[M+H]^+$); 498.702 (7), 498.201 (14), 497.703 (15), 497.201 (22), 496.704 (24), 496.202 (38, $[M+2O+H+Na]^{2+}$); 489.193 (6), 488.697 (6), 488.195 (11, $[M+H+K]^{2+}$); 482.708 (24), 482.207 (49), 481.708 (43), 481.207 (70), 480.71 (64), 480.208 (98, $[M+H+Na]^{2+}$); 470.216 (6), 469.719 (6), 469.217 (12, $[M+2H]^{2+}$) (see Figure S2).

^{1}H NMR (500 MHz, c = 1.1 mM in D_2O, 298K): see main text, Figure 2 and Table S1.

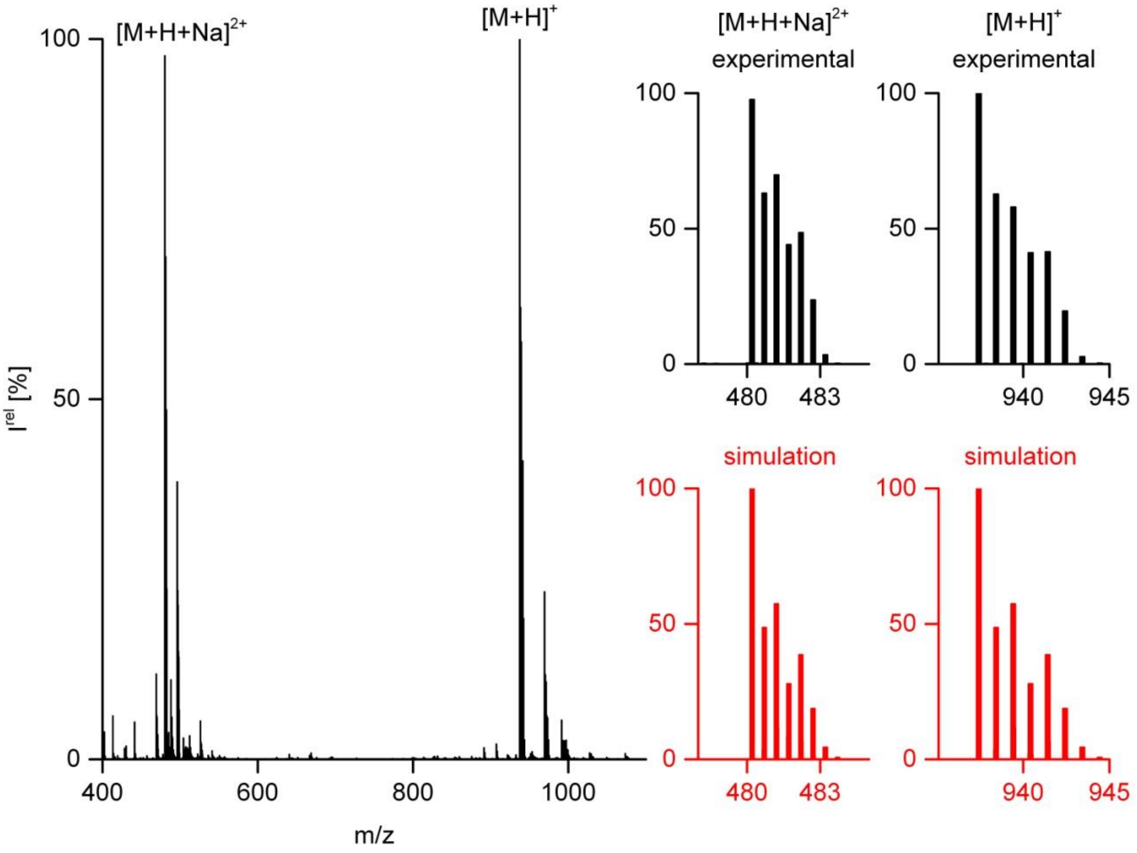

Figure S2. ESI-LTQ-Orbitrap high resolution mass spectrum of **Znby** in MeOH and simulated spectra for the pseudo molecular ions $[M+H+Na]^{2+}$ and $[M+H]^{+}$, spray voltage =4.5kV

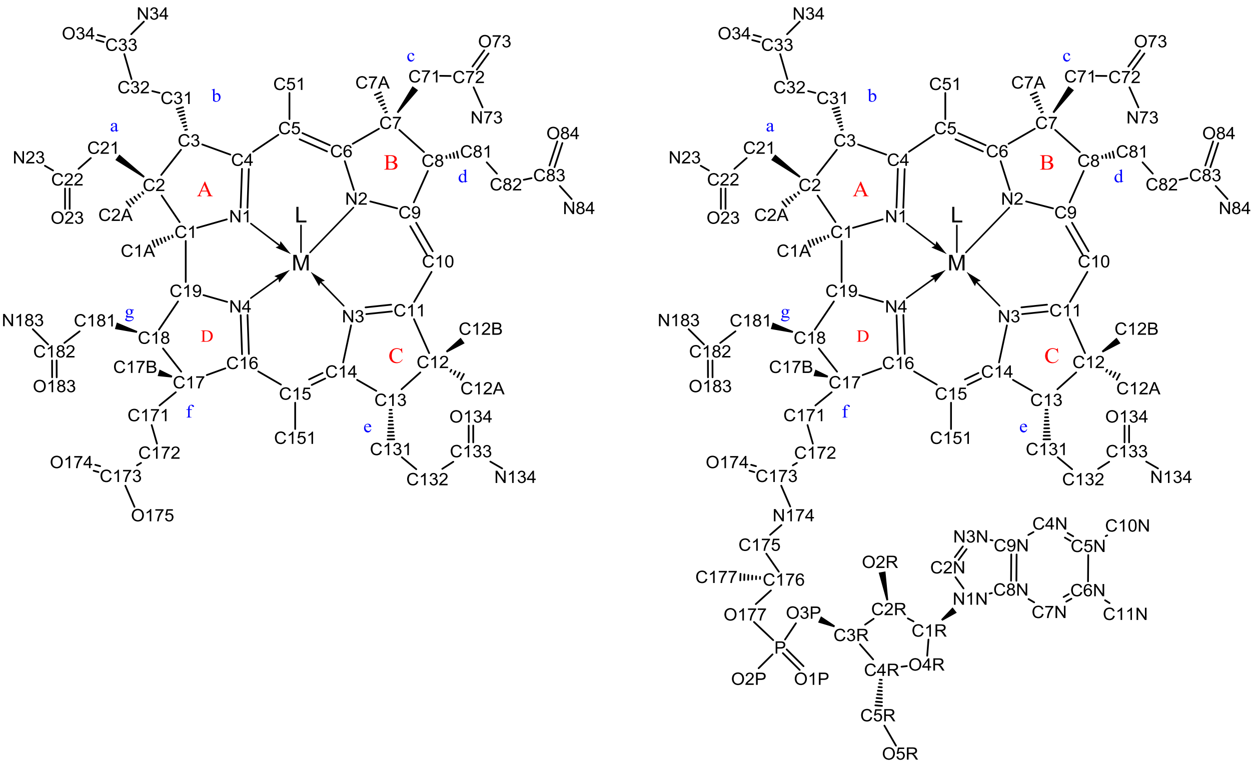

Figure S3. Atom numbering used for **Znby** (left) and **Znbl** (right)

Table S1. ^{1}H and ^{13}C chemical shifts and signal assignment of **Znby** from 500 MHz NMR spectra in D_2O at 298K and shift differences $\Delta\delta=\delta^{Hby}-\delta^{Znby}$ from **Hby**[2] (700MHz, 10mM Na-phosphate pH 5, H_2O/D_2O 49:1, 298K), see Figure S3 for atom numbering.

atom	δ ^{13}C [ppm]	δ ^{1}H [ppm]	multiplicity	*J* [Hz]	Δδ ^{13}C [ppm]	Δδ ^{1}H [ppm]
C1	81.9				-1.3	
C1A	25.5	1.13	s		-4.1	0.11
C2	49.1				-2.8	
C2A	18.8	1.44	s		-3.2	-0.02
C21	45.6	2.34 2.39	AB-system	J_{AB}≈14	-3.1	-0.06 -0.04
C22	179.3				-2.8	
C3	58.8	3.58	d^{app}	8.8	-3.7	-0.11
C31	27.4	2.07 2.17	m		-3.0	-0.23 -0.09
C32	36.0	2.40	m		-1.7	-0.02
C33	179.5[1]				-1.1	
C4	180.6				-2.2	
C5	107.7				-2.4	
C51	17.5	2.18	s		-4.5	-0.04
C6	170.5				-10.5	
C7	51.9				-4.8	
C7A	21.7	1.59	s		-3.1	-0.03
C71	47.7	2.45 2.54	AB-system	J_{AB}=15	-3.4	0.12 0.07
C72	178.3				-3.1	
C8	57.4	3.21	triplettoid	~4.4	-4.0	0.09
C81	28.3	1.83 2.07	m		-3.7	0.08 0.07
C82	34.4	2.24	m		-2.1	0.05
C83	181.0				-2.8	
C9	181.3				-11.1	
C10	91.1	5.51	s		-1.6	0.11
C11	187.5				1.9	
C12	49.7				-1.1	
C12A	20.8	1.36	s		-2.8	-0.01
C12B	33.9	1.17	s		-3.5	-0.02
C13	54.8	3.16	m		-2.2	0.02
C131	27.7	1.79 1.97	m		-3.2	0.00 0.03
C132	37.6	2.40 2.46	m		-6.1	-0.07 -0.04
C133	180.11				-1.7	
C14	169.4				8.2	
C15	106.5				-3.4	
C151	17.8	2.22	s		-4.7	-0.03
C16	182.5				-3.0	
C17	62.9				-5.4	
C17B	21.8	1.32	s		-2.9	0.04
C171	34.9	1.76 2.26	m		-3.2	0.09 -0.27
C172	37.6	2.41	m		-5.0	-0.11
C173	180.9				-0.2	
C18	35.5	2.68	d^{app}		4.0	0.24
C181	42.1	2.75	m		-9.5	-0.01
C182	179.2				-3.0	
C19	74.1	4.18	d	9.5	-7.5	0.14

[1] tentative assignment due to signal overlap

4. Synthesis, purification and spectral analysis of zincobalamin

All operations were performed with protection from light.

In a 5 mL round bottom flask 5.0 mg (4.8 µmol) **Znby**, 6.7mg (16 µmol, 3.3 eq) B_{12}-nucleotide, and 13.1mg (97 µmol, 20eq) 1-hydroxybezotriazole were dissolved in 1 mL H_2O and cooled on ice. A freshly prepared ice cold solution of 21.0 mg (110 µmol, 23eq) EDC*HCl in 800µl H_2O was added to the cold reaction solution. The cooled mixture was stirred for 4 h, diluted to approx. 50 mL with H_2O and loaded on an RP18-MPLC column. The adsorbed **Znbl** was purified using 10 mM NaOAc pH 6 with 0 to 60% MeOH over 10 h. The orange fractions that eluted after approx. 5.5 h were diluted with H_2O to the 2-fold volume and loaded on a SepPak® classic cartridge. The adsorbed **Znbl** was washed with 30 mL H_2O and eluted with ~3ml MeOH: The methanolic solution was evaporated on the rotary evaporator (35 °C) and the residue was dissolved in 50 µL H_2O. The purified **Znbl** was crystallized by the portion wise addition of a total volume of ~3ml MeCN at 5±3°C over 4 days. The crystals were washed with 2x1ml MeCN and dried, yielding 4.8 mg (3.6 µmol, 75%) orange, micro-crystalline **Znbl**.

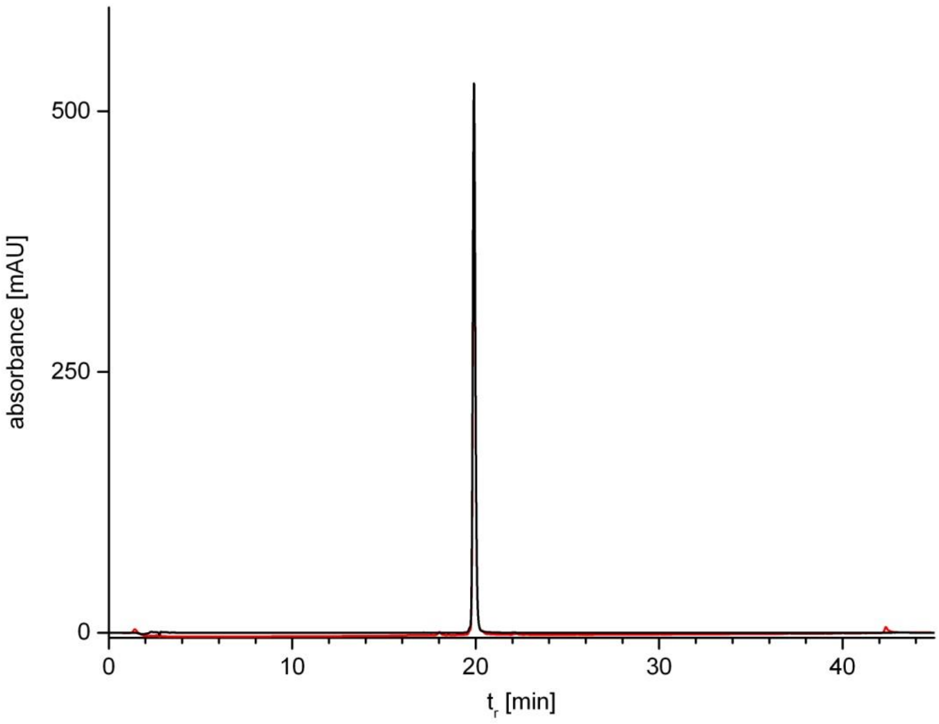

Figure S4. HPLC chromatogram of micro-crystalline **Znbl** (black trace λ=280nm, red trace λ=520nm)

UV/Vis (c=18.2µM in 10mM aq. Na-phosphate buffer pH 5, RT): λ^{max} [nm] (lg ε) = 528 (4.15), 503 (4.16), 478 (sh., 3.98), 359 (sh., 4.00), 339 (4.65), 326 (sh., 4.44), 287 (sh., 4.18), 272 (4.51) (see main text, Figure 1)

CD (c=52.4 µM in 10 mM aq. Na-phosphate-buffer pH 5, 293K): $\lambda^{max/min}$ [nm] ($\Delta\varepsilon$ [l*mol^{-1}*cm^{-1}]) = 526 (-3.5), 502 (-3.5), 423 (-0.1), 337 (10.2), 285 (-1.7), 270 (-0.2), 259 (-0.9), 240 (3.2); λ^0 [nm] = 370, 298, 253 (see main text, Figure 1)

Fluorescence: (c = 8.42 µM in 10 mM aq. Na-phosphate buffer pH 5, RT):
emission (excitation at 505 nm): λ^{max} [nm] (I) = 592 (sh, 125), 560 (171)
excitation spectrum (emission at 560 nm): λ^{max} [nm] (I) = 526 (183), 505 (170), 465 (121), 339 (297), 326 (sh, 216), 278 (222), 271 (214) (see main text, Figure 1)

High resolution ESI-MS (ESI-LTQ-Orbitrap, MeOH, 4.5kV): 1376.519 (15), 1375.521 (15), 1374.519 (20), 1373.524 (19),1372.521 (23, $[M+K]^+$); 1361.548 (18), 1360.546 (31), 1359.549 (35), 1358.547 (50), 1357.550 (45), 1356.548 (55, $[M+Na]^+$); 707.242 (16), 706.741 (23), 706.244 (20), 705.742 (26, $[M+2K]^{2+}$); 700.756 (14), 699.754 (54), 699.255 (54), 698.754 (72), 698.257 (71), 697.756 (95, $[M+Na+K]^{2+}$); 692.769 (12), 692.268 (29), 691.767 (51), 691.268 (49), 690.768 (67), 690.270 (73), 689.769 (100, $[C_{62}H_{88}N_{13}O_{14}PZn+2Na]^{2+}$ ≙ $[M+2Na]^{2+}$); 689.260 (13), 688.759 (19), 688.260 (18), 687.759 (24), 687.262 (25), 686.760 (34, $[C_{61}H_{87}KN_{14}O_{14}PZn]^{2+}$); 678.778 (14, $[M+H+Na]^{2+}$) (see Figure S5)

^{1}H NMR (500 MHz, c = 7.2 mM, D_2O, 298K): (see main text, Figures 2 and S6 and Table S2).

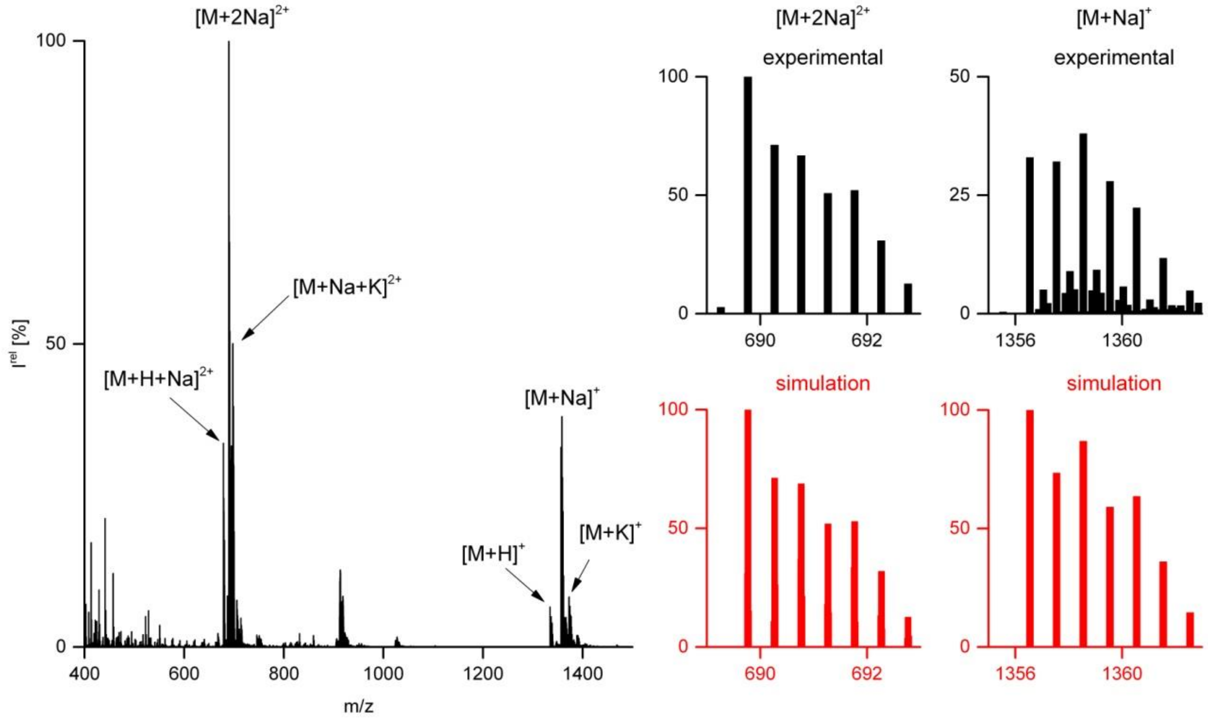

Figure S5. ESI-LTQ-Orbitrap high resolution mass spectrum of **ZnbI** in MeOH and simulated spectra for the pseudo molecular ions $[M+2Na]^{2+}$ and $[M+Na]^+$, spray voltage =4.5kV

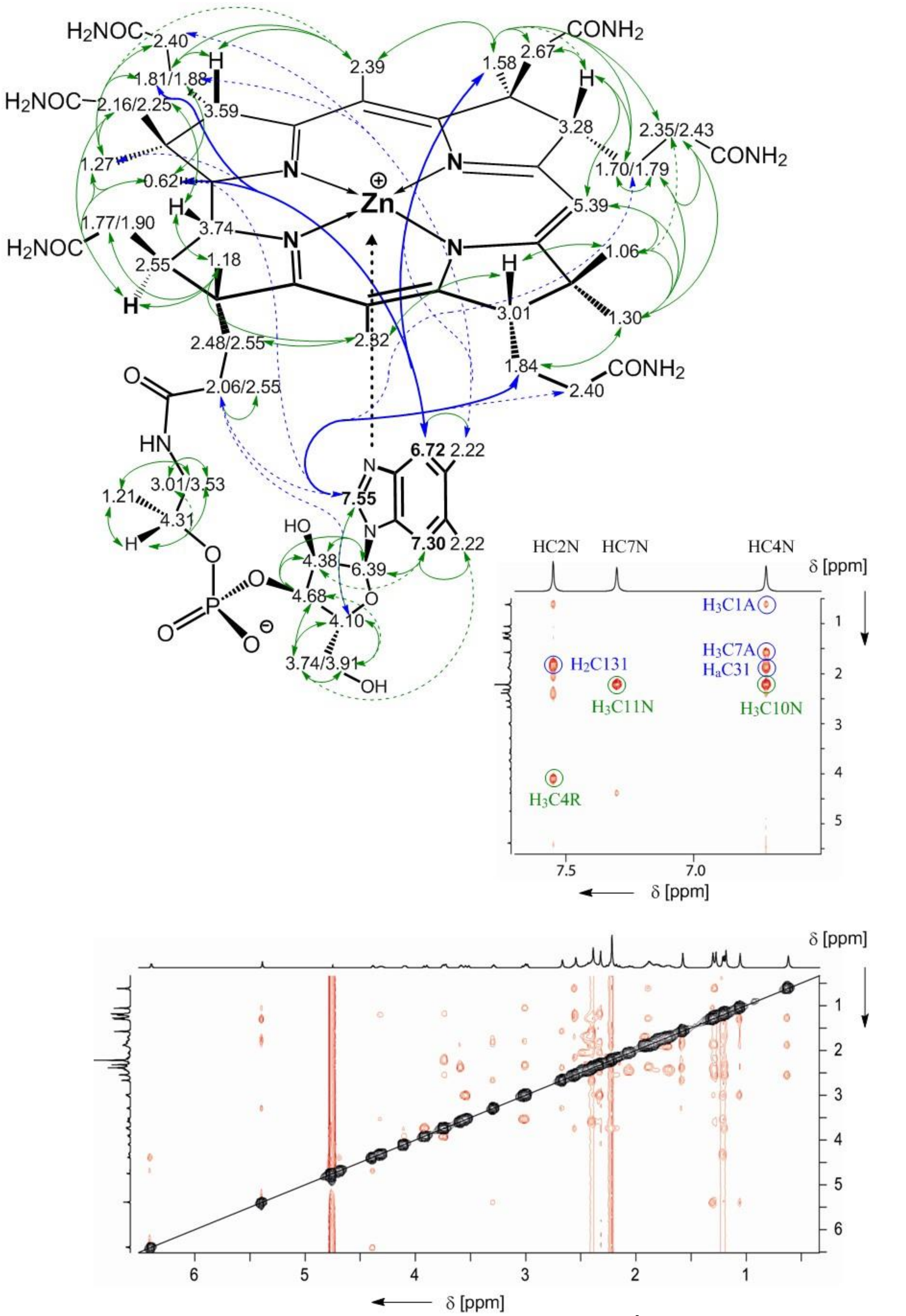

Figure S6. Top, left. “Base-on” solution structure of **Znbl** with assigned ^{1}H chemical shift values (D_2O, 298K), and NOE correlations indicated by arrows (diagnostic DMB-corrin-correlations are colored blue); **right.** low field section of the 500MHz ROESY spectrum of **Znbl** (diagnostic DMB-corrin-correlations are highlighted blue). **Bottom.** high field section of the 500MHz ROESY spectrum of **Znbl.**

Table S2. ^{1}H and ^{13}C chemical shifts and signal assignment of **ZnbI** from 500 MHz NMR spectra in D_2O at 298K, see Figure S3 for atom numbering.

atom	δ ^{13}C	δ ^{1}H [ppm]	multiplicity	*J* [Hz]
C1	80.2			
C1A	20.7	0.62	s	
C2	47.0			
C2A	23.5	1.27	s	
C21	43.0	2.17 2.25	AB-system	J_{AB}=14.0
C22	176.7			
C3	57.8	3.59	t^{app}	4.4
C31	27.6	1.81 1.88	m	
C32	34.1	2.40	m	
C33*	178.0			
C4	178.0			
C5	104.0			
C51	15.5	2.39	s	
C6	169.6			
C7	48.8			
C7A	20.0	1.58	s	
C71	44.2	2.67	triplettoid	~15
C72	175.7			
C8	54.9	3.28	m	
C81	25.3	1.70 1.79	m	
C82	35.1	2.35 2.43	m	
C83	178.0			
C9	179.2			
C10	88.9	5.39	s	
C11	185.1			
C12	47.6			
C12A	19.0	1.30	s	
C12B	30.4	1.06	s	
C13	53.8	3.01		
C131	27.7	1.84	m	
C132	34.6	2.4	m	
C133	178.0			
C14	175.2			
C15	104.3			
C151	15.3	2.32	s	
C16	179.2			
C17	58.8			
C17B	16.7	1.18	s	
C171	32.4	2.48 2.55	m	
C172	36.3	2.06 2.55	m	
C173	175.3			
C175	45.0	3.01 3.53	H_B=d	*J*(H_B)=13.9Hz
C176	72.8	4.31	q	6.6
C177	18.7	1.21	d	6.6
C18	40.5	2.55		
C181	32.6	1.77 1.90	m	
C182	177.7		m	
C19	71.3	3.74		
C1R	86.6	6.39	d	2.9
C2R	68.9	4.38	t^{app}	3.7
C3R	72.9	4.68		
C4R	81.6	4.10	m	
C5R	60.3	3.74 3.91	H_B=d	*J*(H_B)=11.7
C2N	141.3	7.55	s	
C4N	117.4	6.72	s	
C5N	134.8			
C6N	133.5			
C7N	110.8	7.30	s	
C8N	137.1			
C9N	129.8			
C10N	19.5	2.22	s	
C11N	19.5	2.22	s	

5. Crystallization of zincobyric acid and single-crystal X-ray structure analysis

Single crystals of **Znby** were grown from H_2O/MeCN containing ~10 µM $NaBF_4$ at 5±3°C. Data of a single crystal of **Znby** were collected at 173K with a Bruker D8 Quest diffractometer (Photon 100 detector) equipped with a microfocus source generator combined with multi-layer optics (monochromatized Mo K_α radiation, λ = 71.073 pm). The structure was solved with SHELXT[3] (version 2014/4) and structure refinement (full-matrix least-squares against F^2) with SHELXL[4] (version 2014/7). Relevant details of the data collection and data evaluation are listed in Table S3 (see main text, Figures 3 and 4, and Figure S3)

Supporting crystallographic data of **Znby** may be obtained from the Cambridge Crystallographic Data Centre CCDC deposition service *via* www.ccdc.cam.ac.uk/structures on quoting the deposition number CCDC 1921462.

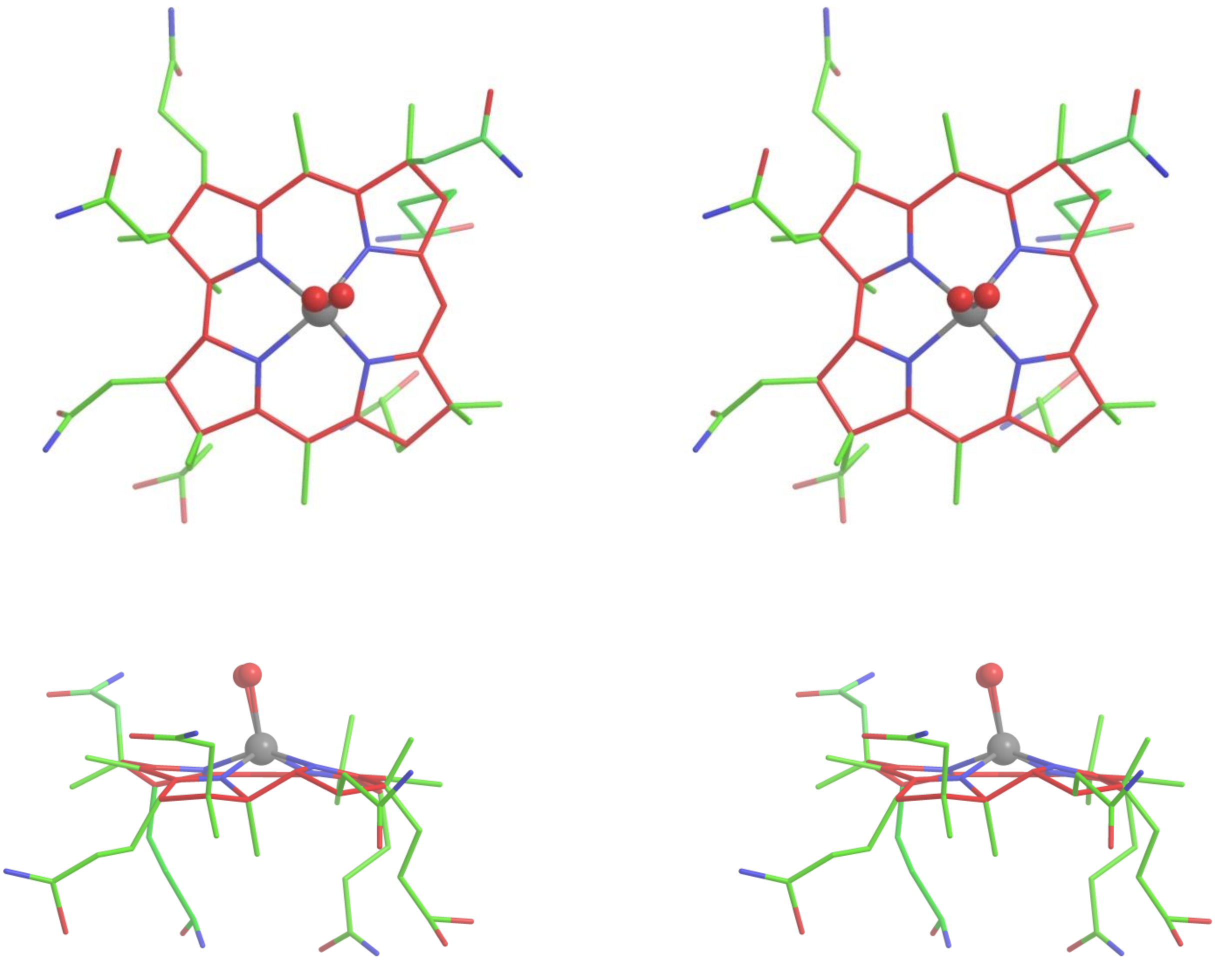

Figure S7. X-ray crystal structure: Stereo-pictures of the molecular structure of **Znby** highlighting the disordered coordinating O-atoms of the adjacent **Znby** molecule.

Table S3. Crystal data, structure refinement of **Znby** (CCDC 1921462)

Empirical formula	$C_{45}H_{64}N_{10}O_8Zn$ x 13 H_2O	
Formula weight	1172.63	
Temperature	173(2) K	
Wavelength	0.71073 Å	
Crystal system	Orthorhombic	
Space group	$P2_12_12_1$ (no. 19)	
Unit cell dimensions	a = 14.4736(10) Å	α = 90°
	b = 17.5252(12) Å	β = 90°
	c = 24.3115(16) Å	γ = 90°.
Volume	6166.7(7) $Å^3$	
Z	4	
Density (calculated)	1.263 Mg/m^3	
Absorption coefficient	0.474 mm^{-1}	
F(000)	2512	
Crystal size	0.160 x 0.110 x 0.070 mm^3	
Theta range for data collection	2.324 to 19.796°	
Index ranges	-13<=h<=13, -16<=k<=16, -23<=l<=23	
Reflections collected	32624	
Independent reflections	5566 [R(int) = 0.0901]	
Completeness to theta = 19.796°	99.5 %	
Absorption correction	Semi-empirical from equivalents	
Max. and min. transmission	0.942 and 0.850	
Refinement method	Full-matrix least-squares on F^2	
Data / restraints / parameters	5566 / 22 / 657	
Goodness-of-fit on F^2	1.076	
Final R indices [I>2sigma(I)]	R1 = 0.0778, wR2 = 0.1927	
R indices (all data)	R1 = 0.1033, wR2 = 0.2105	
Absolute structure parameter	0.031(9)	
Extinction coefficient	n/a	
Largest diff. peak and hole	0.377 and -0.311 e.$Å^{-3}$	

Table S4. Experimental structural data of **Znby** and comparison with structural data from **Hby**[2] and **Cbin**^{II}[3]. d: distance between atoms [Å]; δ:distance of atom from a best plane [Å]; τ: torsion angle [°]; corrin fold angle: angle between the best planes A (N1, C4, C5, C6, N2, C9, C10) and B (C10, C11, N3, C14, C15, C16, N4) [°]; *h*: corrin helicity, dihedral angle (N1-N2-N3-N4) around a virtual bond between N2 and N3 [2]; ϕ: angle between the planes (N1-metal center-N4) and (N2-metal center-N3)[2] [°]; ψ: angle between the planes (N1-metal center-N2) and (N3-metal center-N4) [°]; p1: best plane through (N1-N2-N3-N4); p2: best plane through (C1-C4-C6-C9-C11-C14-C16-C19); p3: best plane through (C2-C3-C5-C7-C8-C10-C12-C13-C15-C17-C18) (see illustrations below)

		Znby	Hby	Cbin^{II}
d	M-N1	2.039	1.760	1.889
	M-N2	2.031	2.051	1.931
	M-N3	1.986	1.923	1.906
	M-N4	2.054	1.930	1.874
	M-L	2.058/2.095	-	2.312
	N1-N2	2.719	2.620	2.676
	N2-N3	2.853	2.996	2.867
	N3-N4	2.770	2.665	2.667
	N4-N1	2.571	2.608	2.538
	N1-N3	3.751	3.668	3.782
	N2-N4	3.937	3.965	3.799
δ	M-p1	+0.624	-	+0.05
	N1-p1	-0.107	-0.172	-0.079
	N2-p1	+0.096	+0.149	+0.070
	N3-p1	-0.094	-0.144	-0.070
	N4-p1	+0.104	+0.167	+0.079
	M-p2	+0.790	-	+0.030
	N1-p2	+0.062	-0.093	-0.091
	N2-p2	+0.137	+0.094	-0.021
	N3-p2	+0.084	-0.050	-0.085
	N4-p2	+0.398	+0.378	+0.135
	M-p3	+0.927	-	-0.044
	N1-p3	+0.202	-0.034	-0.193
	N2-p3	+0.234	+0.127	-0.135
	N3-p3	+0.225	+0.033	-0.127
	N4-p3	+0.577	+0.481	+0.096
	N1-(M-C1-C4)	0.001	-	+0.054
	N2-(M-C6-C9)	-0.144	-	-0.090
	N3-(M-C11-C14)	-0.102	-	-0.064
	N4-(M-C16-C19)	+0.038	-	+0.033
τ	N1-C4-C5-C6	-17.37	-16.04	-7.09
	N4-C16-C15-C14	-14.03	-5.60	-0.80
corrin fold angle θ		6.38	11.64	6.01
helicity *h*		7.97	12.92	6.07
interplanar angle ϕ		50.2	-	7.56
interplanar angle ψ		50.31	-	7.56

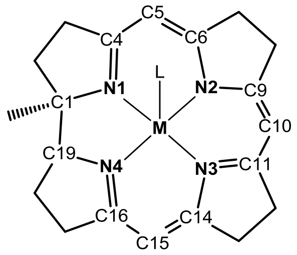

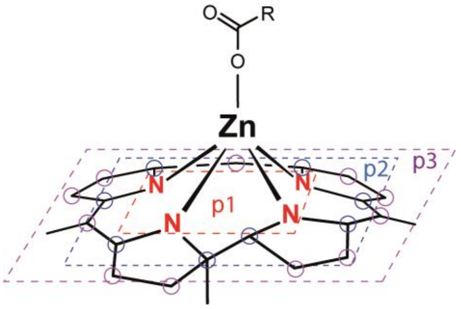

corrin fold angle

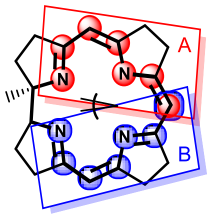

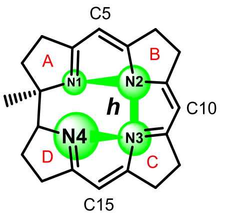

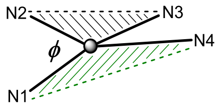

6. Metal incorporation into hydrogenobyric acid – kinetic analysis

6.1. Zinc insertion kinetics

20 µL of a 4.19 mM **Hby** stock solution were diluted with 140 µL H_2O and the metallation reaction was initiated by the addition of 40 µL 0.50 M $Zn(OAc)_2$ pH 5. The reaction progress at 21-24 °C was monitored by HPLC analysis.

HPLC analysis: 20 µL of the reaction solution were diluted with 180 µL H_2O and 100 µL of this solution were injected into the Dionex 3000 system and analyzed using 8-95% MeOH in 10 mM NH_4OAc aq. in 40 min. The reaction progress was determined by the relative **Znby** peak area at λ = 520 nm (A_{rel}^{520nm}).

6.1. Cobalt insertion kinetics

20 µL of a 4.19 mM **Hby** stock solution was diluted with 140 µL H_2O and the metallation reaction was initiated by the addition of 40 µL 0.50 M $Co(OAc)_2$ pH 5. The reaction progress at 21-24 °C was monitored by HPLC analysis.

HPLC analysis: To 20 µL of the reaction solution 100 µL aqueous 0.1 M KCN were added. The sample solution was diluted with H_2O to approx. 500 µL, injected into the Hitachi system and analyzed as dicyano cobyric acid using 8-95% MeOH in 10 mM NH_4OAc aq. containing 50 µM KCN in 40 min. The reaction progress was determined by the relative dicyano cobyric acid peak area at λ = 520nm (A_{rel}^{520nm}).

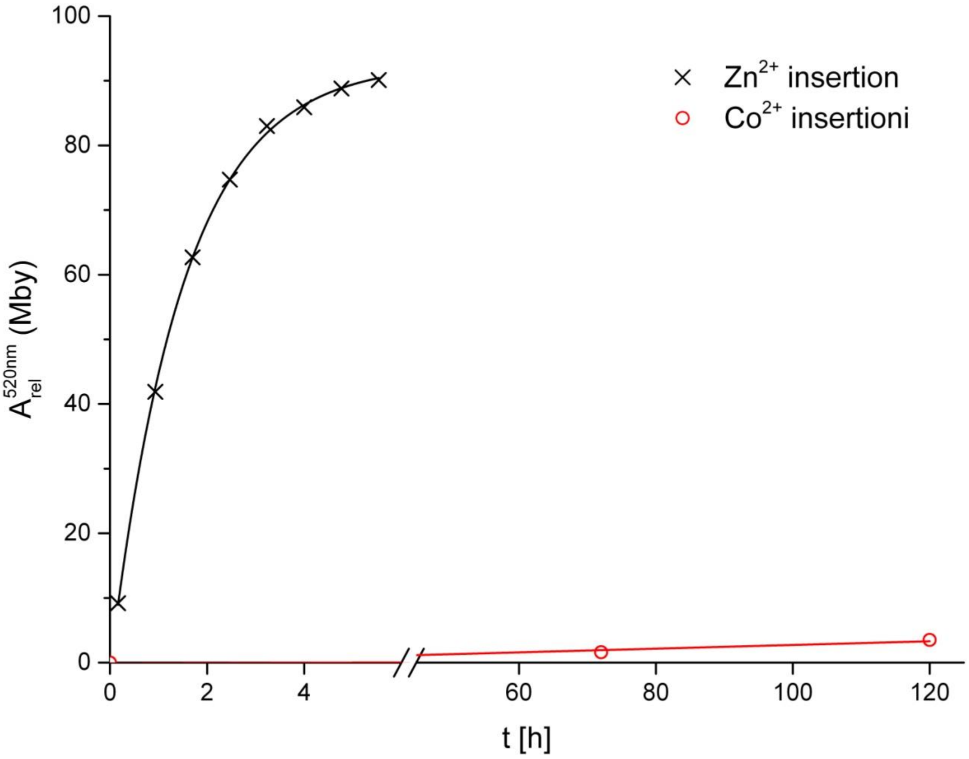

Figure S8: Kinetic comparison of Zn^{2+}(black) versus Co^{2+} (red) insertion into **Hby** at c=0.42mM **Hby** in 100mM $Zn(OAc)_2$ or $Co(OAc)_2$ pH 5

7. Photo-physical measurements of zincobyric acid

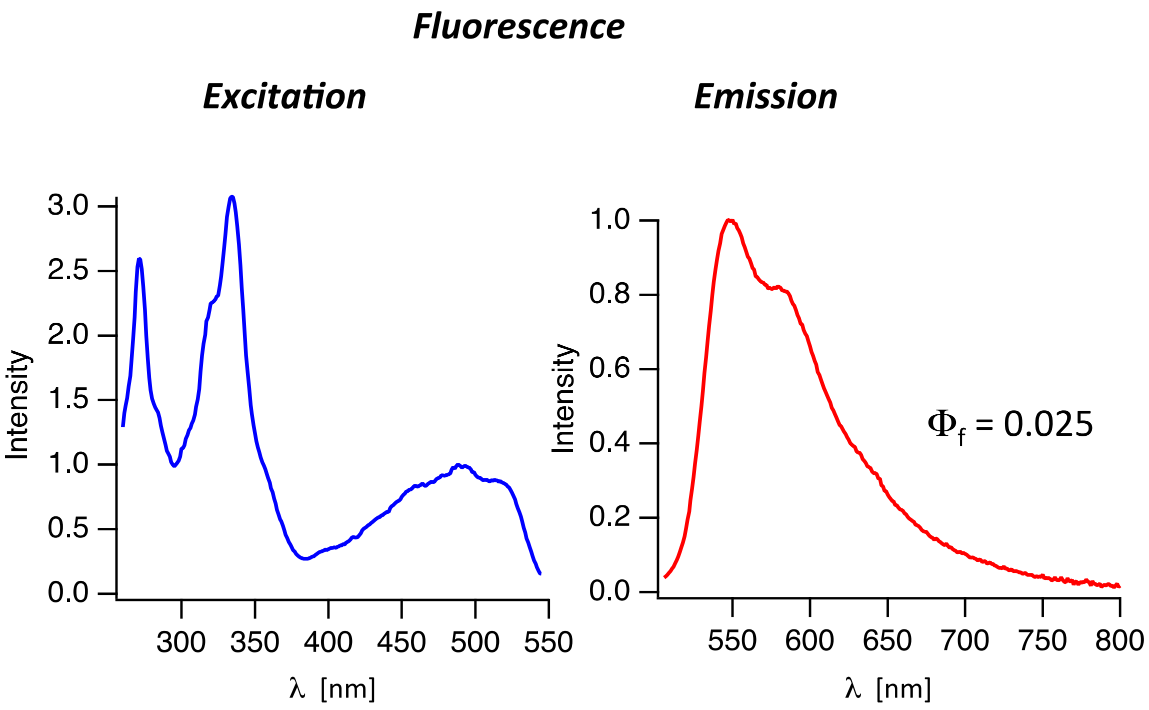

Figure S9. Singlet excited state properties of **Znby**: Fluorescence excitation and emission spectra of **Znby** (c = 8 μM) in ethanol at room temperature. Left: excitation spectrum monitored at λem = 550 nm. Right: fluorescence emission spectrum, λex = 500 nm. The fluorescence quantum yield of **Znby** (Φ_f = 0.025) was determined in reference to rhodamine 6G (Φ_f^{Rh6G} = 0.94) [5].

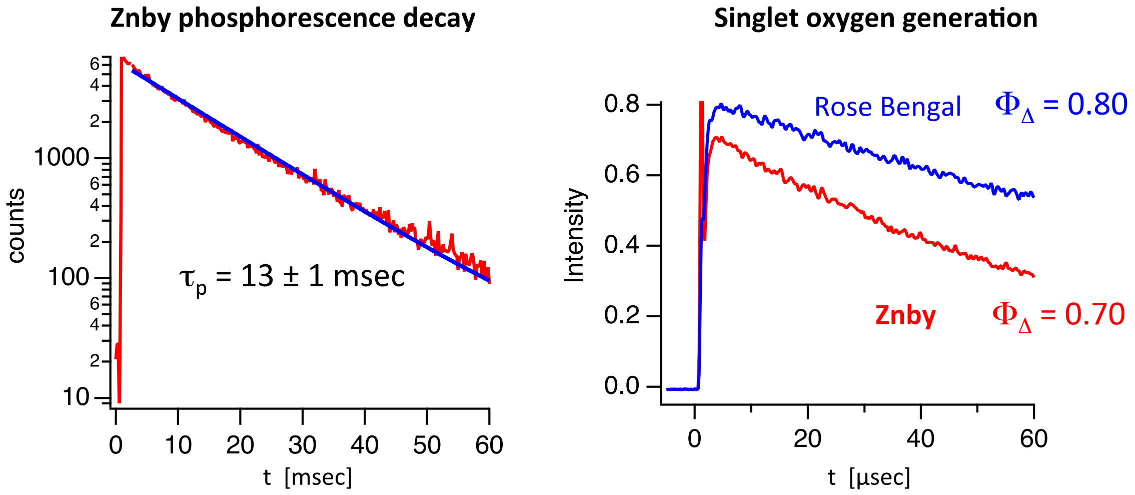

Figure S10. Triplet state properties of **Znby**: Left. phosphorescence decay trace of **Znby** (red line) in ethanol glass at 77K measured by multi-channel scaling (λ_{ex} = 523 nm) monitored at λ_{em} = 628 nm. The mono-exponential fit is shown in blue. Right. singlet oxygen phosphorescence decay traces monitored at 1270 nm after pulsed excitation (λ_{ex} = 532 nm, 7 ns pulse length) of air saturated d_6-ethanol solutions of **Znby** (red) and the reference, Rose Bengal (blue). The quantum yield of singlet oxygen generation (Φ_Δ) by Znby was determined using the known quantum yield of the standard, Rose Bengal (Φ_Δ=0.80)[6].

8. Computational structure calculations of zincobyric acid

As previous studies showed that the conformations of the amide side chains may differ from the crystal structure, and are susceptible to intramolecular H-bonding, the previously published optimized gas phase structure of **Hby** [2] was taken as a starting structure to obtain consistent results. The two inner hydrogen atoms were removed and replaced by Zn^{2+} and the resulting structure on **Znby(4)** was geometry optimized using Density Functional Theory. To avoid further issues with intramolecular hydrogen bonding, the X-ray crystal structure of Co(II)-heptamethyl-cobyrinate perchlorate (Co^{II}-cobester, **Cbin^{II}**)[7] was taken as an alternative starting structure and modified by replacing Co(II) by Zn(II), giving **Znby(4)^{Cob}**. Subsequent structure optimization revealed the geometry of the corrin ligand was very similar to the one of **Znby(4)**. To assess the effect of coordination of the 5th ligand in **Znby** on the structure of the corrin ring and the coordination geometry of Zn, three models were investigated: (i) **Znby^{Cob}**, where the coordination on the β-face is modelled with an acetate ligand, (ii) **Znby(α)^{Cob}**, where an acetate ligand is coordinated on the α-face, and (iii) **Znby(4)^{Cob}**, where Zn is only 4-fold coordinated and the ligand on the β-face is omitted. To determine the lowest energy conformation of the acetate ligand, a dihedral scan was performed for **Znby^{Cob}** and **Znby(α)^{Cob}**, where the structures were allowed to fully relax and only the dihedral angle under study was fixed. In both cases, the acetate ligand prefers and east-west orientation. However, several low energy conformations could be determined in both cases. For structural comparison, the core structures of **Znby^{Cob}** and **Hby**, were aligned on the respective corrin ring atoms.

The **Znbl** structure was obtained by modification of a previously optimized gas phase structure of **Cbl^{II}** derived from the X-ray crystal structure of an antivitamin B_{12}.[8]

All calculations were performed with the quantum chemical suite Turbomole.[9] A def2-TZVP basis set was used for all atoms.[10] In order to speed up calculation time the resolution-of-identify (RI) technique was utilized.[11] To critically assess the performance of density functionals, both, the BP86[12] and the PBE[13], density functional were used for structure optimizations together with empirical dispersion corrections of the D3 type with Becke-Johnson damping (BJ).[14] However, structural differences were found to be minimal.

All structures were visualized with PyMol.[15]

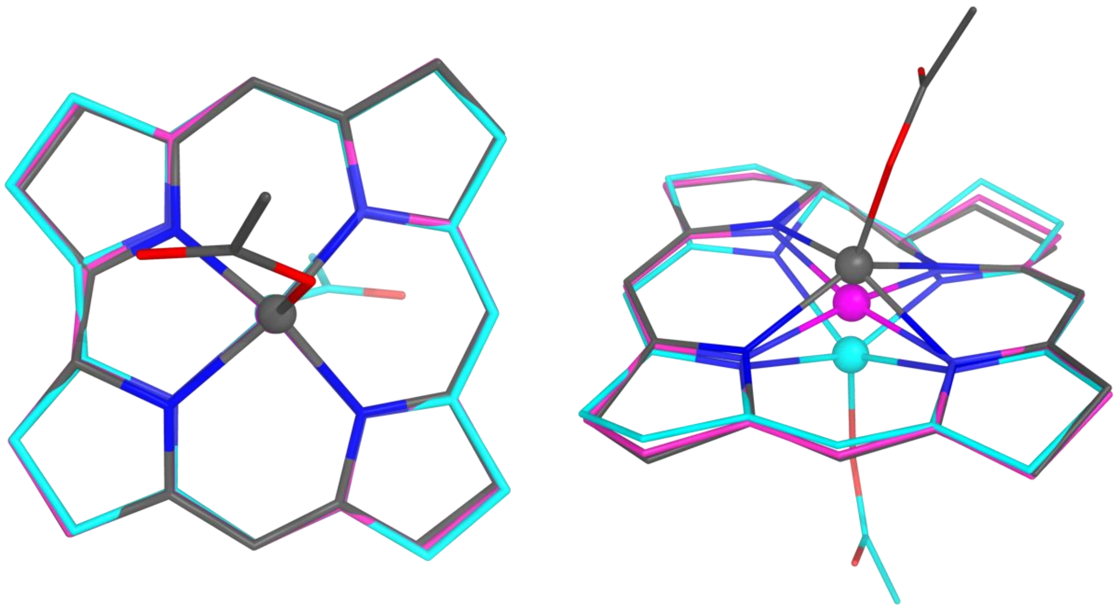

Figure S11. Overlay of calculated zincobyrate structures (left: top view; right: east view) when optimized with BP86/def2-TZVP/D3 in gas phase. Gray: **Znby**$^{\mathbf{Cob}}$ structure with acetate coordinated on the β-face. Turquoise: **Znby(α)**$^{\mathbf{Cob}}$ structure with acetate coordinated on the α-face. Magenta: **Znby(4)**$^{\mathbf{Cob}}$ structure with 4-coordinated Zn(II)-center.

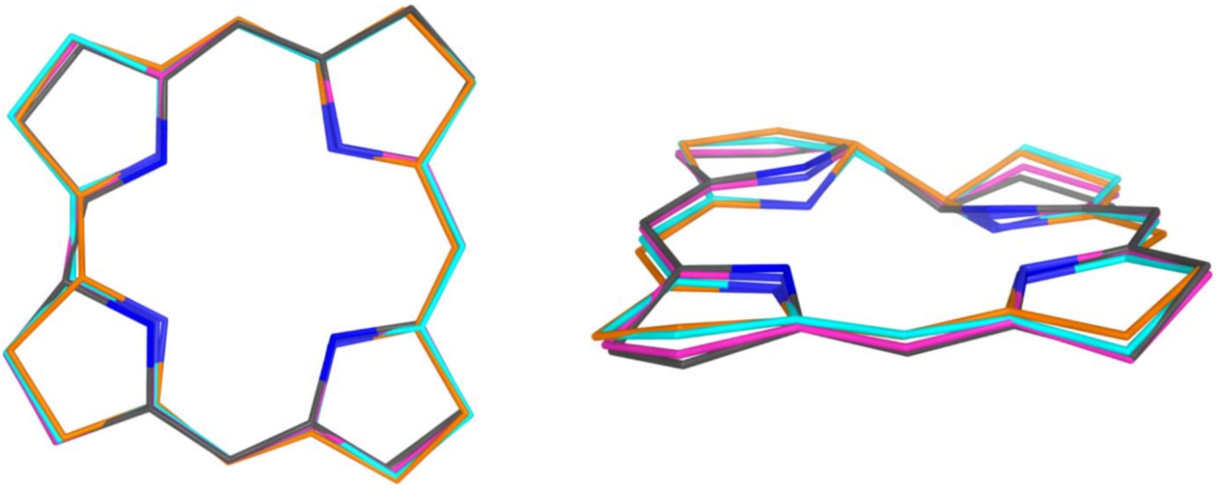

Figure S12: Overlay of calculated zincobyrate structures with **Hby** when the metal centers were omitted for clarity (left: top view; right: east view). Structures were optimized with BP86/def2-TZVP/D3 in gas phase. Gray: **Znby**$^{\mathbf{Cob}}$ structure with acetate coordinated from above. Turquoise: **Znby(α)**$^{\mathbf{Cob}}$ structure with acetate coordinated from below. Magenta: **Znby(4)**$^{\mathbf{Cob}}$ structure with 4-coordinated Zn(II)-center. Orange: **Hby** structure.

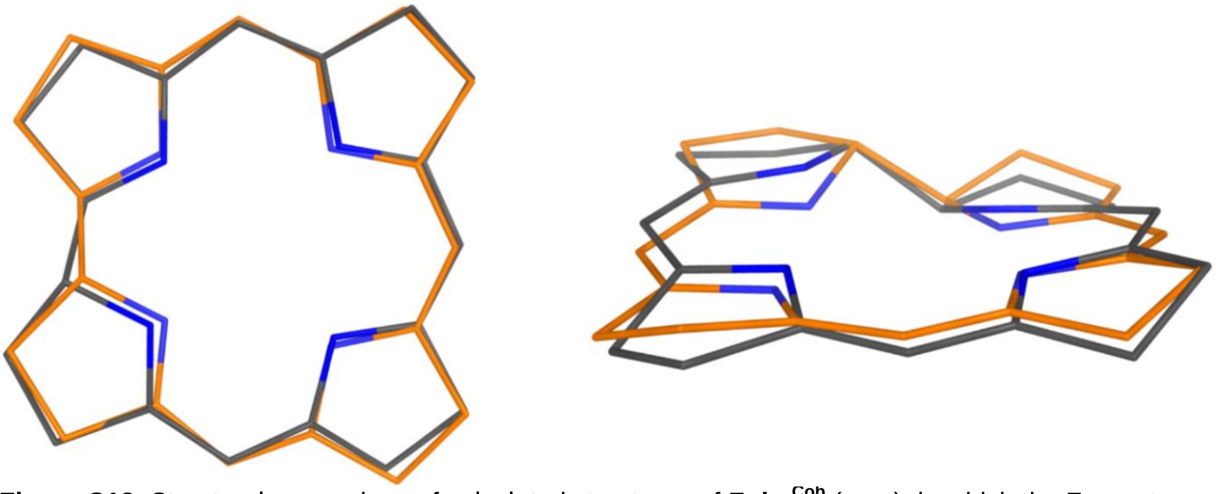

Figure S13. Structural comparison of calculated structures of **Znby**^{Cob} (gray), in which the Zn-center was omitted for clarity, with **Hby** (orange) (left: top view; right: east view). Structures were optimized with BP86/def2-TZVP/D3 in gas phase.

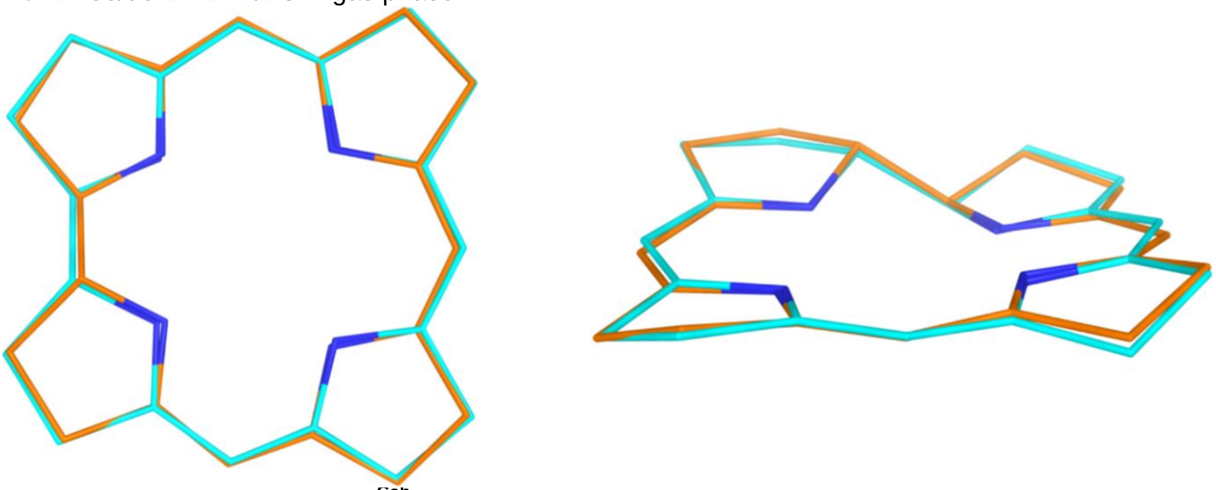

Figure S14. Overlay of **Znby(α)**^{Cob} (turquoise), in which the Zn-center was omitted for clarity, with **Hby** (orange) (left: top view; right: east view). Structures were optimized with BP86/def2-TZVP/D3 in gas phase.

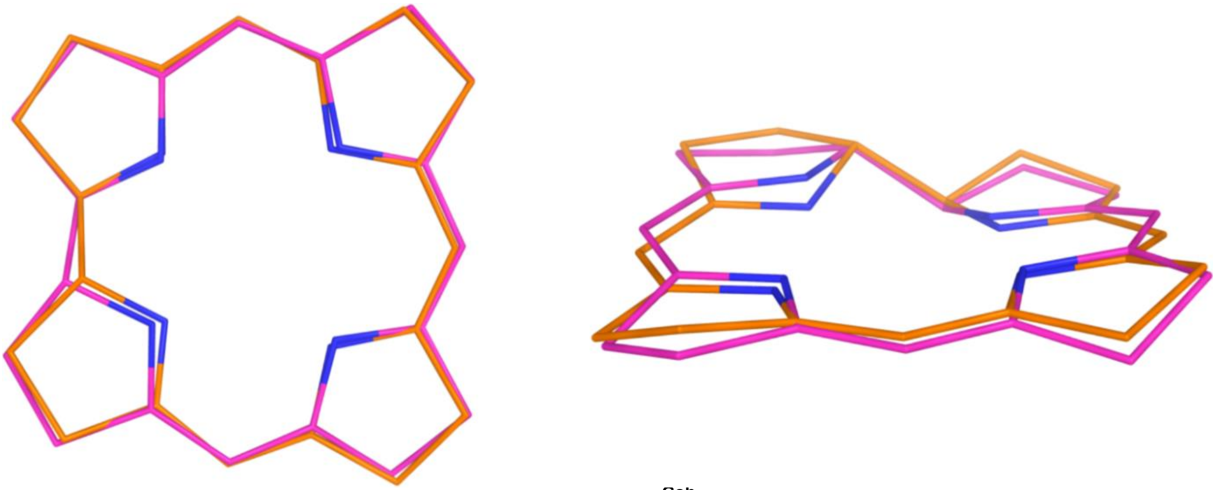

Figure S15 Overlay of calculated structures of **Znby(4)**^{Cob} (magenta), in which the 4-coordinated Zn-center was omitted for clarity, with **Hby** (orange) (left: top view; right: east view). Structures were optimized with BP86/def2-TZVP/D3 in gas phase.

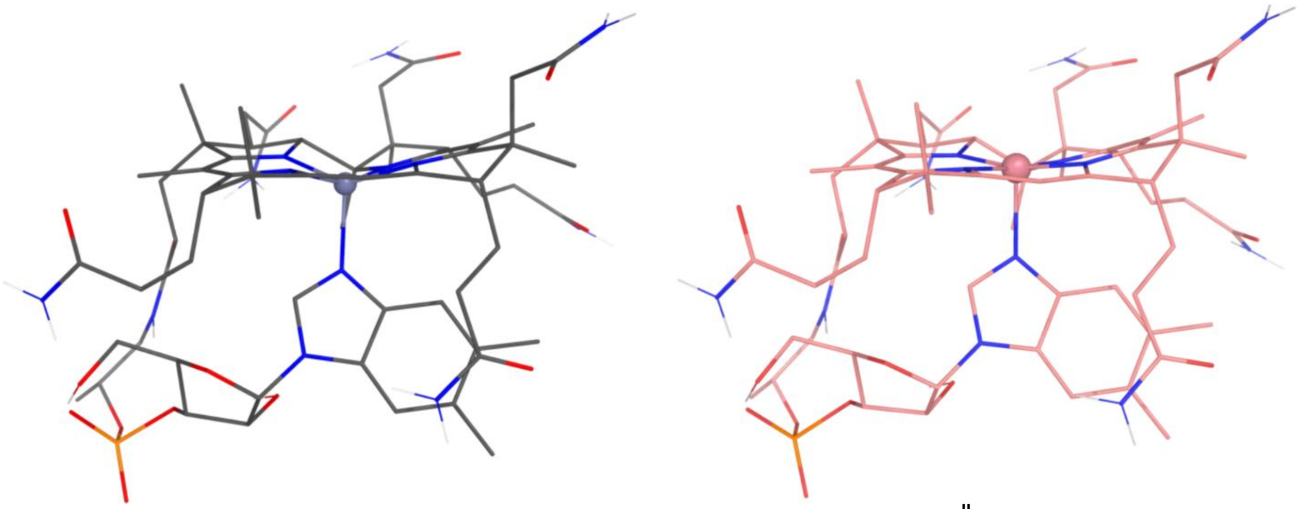

Figure S16. Quantum chemically optimized structures of **Znbl** (left) and **Cbl^{II}** (pink) (BP86/def2-TZVP/D3)

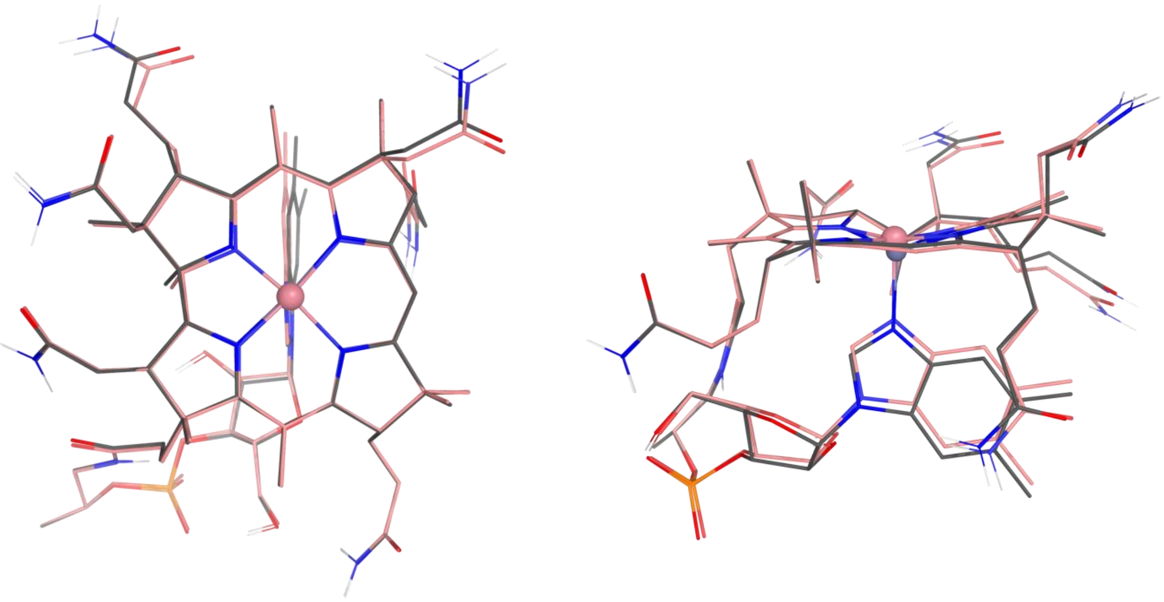

Figure S17: Overlay of quantum chemically optimized structures **Cbl^{II}** (pink) and **Znbl** (gray) (left top view, right east view).

Table S4. Calculated structural parameters in Å and ° of the optimized structures of Zn-corrins with 4-fold coordination of Zn, **Znby(4)** and **Znby(4)^{cob}** as well as 5-fold coordinated **Znby^{Cob}** and **Znby(α)^{Cob}**, in which acetate coordinates at the Zn-center from above or below, respectively. Prior to the evaluation of the 5-fold coordinated Zn-analogue of cobester a dihedral scan of the acetate group was performed to determine the energetically most favorable position.

	**Znby(4)**		**Znby(4)^{cob}**		**Znby^{Cob}**		**Znby(α)^{Cob}**	
	BP86/D3	PBE/D3	BP86/D3	PBE/D3	BP86/D3	PBE/D3	BP86/D3	PBE/D3
N1-N2	2.81	2.81	2.79	2.81	2.72	2.73	2.81	2.82
N2-N3	2.96	2.96	2.95	2.97	2.94	2.94	2.95	2.96
N3-N4	2.82	2.83	2.84	2.82	2.82	2.82	2.77	2.77
N1-N4	2.59	2.59	2.58	2.59	2.57	2.58	2.57	2.58
N1-N3	3.90	3.90	3.88	3.91	3.77	3.78	3.95	3.96
N2-N4	3.93	3.95	3.95	3.93	3.99	4.00	3.86	3.86
Zn-N1	1.97	1.97	1.97	1.98	2.05	2.06	2.05	2.06
Zn-N2	1.98	1.98	1.97	1.97	2.10	2.10	2.05	2.06
Zn-N3	1.95	1.96	1.95	1.96	2.04	2.05	2.04	2.05
Zn-N4	1.99	1.99	1.99	1.99	2.05	2.05	2.08	2.09
Zn-OAc	-	-	-	-	1.99	2.00	2.02	2.02
N1-C4	1.30	1.30	1.30	1.30	1.29	1.29	1.30	1.30
C4-C5	1.46	1.46	1.46	1.46	1.46	1.46	1.45	1.45
C5-C6	1.39	1.39	1.39	1.39	1.38	1.38	1.39	1.39
C6-N2	1.39	1.39	1.39	1.38	1.38	1.38	1.38	1.37
N2-C9	1.35	1.35	1.35	1.35	1.33	1.33	1.35	1.35
C9-C10	1.40	1.40	1.40	1.40	1.41	1.41	1.40	1.40
C10-C11	1.40	1.40	1.40	1.40	1.39	1.39	1.40	1.40
C11-N3	1.35	1.35	1.35	1.35	1.35	1.35	1.34	1.34
N3-C14	1.38	1.38	1.38	1.38	1.38	1.37	1.38	1.38
C14-C15	1.39	1.39	1.39	1.39	1.39	1.39	1.39	1.39
C15-C16	1.45	1.46	1.46	1.46	1.45	1.46	1.45	1.46
C16-N4	1.31	1.31	1.31	1.31	1.30	1.30	1.30	1.30
N4-C19	1.47	1.47	1.47	1.47	1.46	1.46	1.47	1.47
C19-C1	1.59	1.60	1.60	1.60	1.59	1.59	1.59	1.59
C1-N1	1.47	1.47	1.47	1.47	1.46	1.46	1.47	1.47
$\theta_{N1\text{-}N2\text{-}N3\text{-}N4/}$ *h*	13.4	13.4	13.2	12.7	10.6	10.4	9.0	8.8
$\phi_{interplanar}$	14.7	14.8	16.3	13.9	52.9	54.0	48.5	49.7
$d_{M\ to\ plane}$	0.01	0.02	0.09	0.01	0.66	0.68	-0.61	-0.63
ΔE [kJ/mol]					0.0	0.0	3.1	5.9

Table S5. Calculated structural parameters in Å and in ° of the optimized structure of **ZnbI** and **Cbl^{II}** and comparison with experimental single crystal X-ray data of **Cbl^{II}** [16].

	**ZnbI** BP86/D3	**Cbl^{II}** BP86/D3	**Cbl^{II}-experimental**
N1-N2	2.82	2.68	2.68
N2-N3	2.95	2.83	2.81
N3-N4	2.80	2.69	2.69
N1-N4	2.58	2.47	2.48
N1-N3	3.97	3.77	3.76
N2-N4	3.88	3.76	3.76
M-N1	2.00	1.86	1.87
N-N2	2.02	1.92	1.91
M-N3	2.03	1.92	1.89
M-N4	2.03	1.87	1.87
$M\text{-}N_{Benz}$	2.07	2.11	2.13
N1-C4	1.30	1.31	1.30
C4-C5	1.46	1.44	1.43
C5-C6	1.39	1.38	1.36
C6-N2	1.38	1.39	1.40
N2-C9	1.35	1.35	1.34
C9-C10	1.40	1.39	1.34
C10-C11	1.40	1.39	1.41
C11-N3	1.35	1.36	1.34
N3-C14	1.38	1.39	1.41
C14-C15	1.39	1.38	1.40
C15-C16	1.46	1.43	1.45
C16-N4	1.30	1.32	1.32
N4-C19	1.47	1.49	1.49
C19-C1	1.59	1.55	1.54
C1-N1	1.47	1.50	1.47
$\theta_{N1\text{-}N2\text{-}N3\text{-}N4/}\,h$	8.3	6.0	5.9
$\phi_{\text{interplanar angle}}$	37.5	13.0	12.0
$\delta_{\text{M to plane}}$	-0.46	-0.13	-0.12
$\theta_{\text{corrin fold angle}}$	11.1	13.7	16.2

9. References

[1] a) A. Eschenmoser, *Angew. Chem. Int. Ed.* **1988**, *27*, 5; b) F. Kreppelt, ETH Zürich, doi: 10.3929/ethz-a-000626280 (Zürich), **1991**.

[2] C. Kieninger, E. Deery, A. D. Lawrence, M. Podewitz, K. Wurst, E. Nemoto-Smith, F. J. Widner, J. A. Baker, S. Jockusch, C. R. Kreutz, K. R. Liedl, K. Gruber, M. J. Warren, B. Kräutler, *Angew. Chem. Int. Ed.* **2019**, *58*, 10756.

[3] G.M. Sheldrick. SHELXT - Integrated space-group and crystal-structure determination. *Acta Cryst. Section A* 2015, **71**(1)**:** 3-8.

[4] G.M. Sheldrick. Crystal structure refinement with SHELXL. *Acta Cryst. Section C, Structural chemistry* 2015, **71**(Pt 1)**:** 3-8.

[5] M. Fischer, J. Georges, *Chem. Phys. Lett.* **1996**, *260*, 115-118.

[6] F. Wilkinson, W. P. Helman, A. B. Ross, *J. Phys. Chem. Ref. Data*, **1993**, *22*, 113-262

[7] B. Kräutler, W. Keller, M. Hughes, C. Caderas, C. Kratky, *J. Chem. Soc., Chem. Comm.* **1987**, 1678.

[8] M. Ruetz, C. Gherasim, K. Gruber, S. Fedosov, R. Banerjee, B. Kräutler, *Angew. Chem. Int. Ed.* **2013**, *52*, 2606.

[9] R. Ahlrichs, M. Bär, M. Häser, H. Horn, C. Kölmel, *Chem. Phys. Lett.* **1989**, *162*, 165.

[10] F. Weigend, R. Ahlrichs, *Phys. Chem. Chem. Phys.* **2005**, *7*, 3297.

[11] K. Eichkorn, O. Treutler, H. Ohm, M. Haser, R. Ahlrichs, *Chem. Phys. Lett.* **1995**, *240*, 283.

[12] a)J. P. Perdew, *Physical Review B* **1986**, *33*, 8822; b)A. D. Becke, *Physical Review A* **1988**, *38*, 3098.

[13] J. P. Perdew, K. Burke, M. Ernzerhof, *Phys. Rev. Lett.* **1996**, *77*, 3865.

[14] S. Grimme, S. Ehrlich, L. Goerigk, *J. Comput. Chem.* **2011**, *32*, 1456.

[15] The PyMOL Molecular Graphics System, Version 1.8. **2015**.

[16] B. Kräutler, W. Keller, C. Kratky, *J. Am. Chem. Soc.* **1989**, *111*, 8936.
